# The I. H. A. Meeting at Niagara

**Published:** 1888-08

**Authors:** 


### THE I. H. A. MEETING AT NIAGARA.

The work done was never so great as at the Niagara meeting. The new "*Bureau of Homœopathics*" occupied the first day, and papers have gone on record that will stand as reference texts so long as life is found on this mundane sphere.

There was no sneering at statements made that could not at first be considered of facts, but, on the contrary, thoughtful faces were seen everywhere. They listened and reflected over the proofs that formed opinions; they listened to the verified symptoms, to the confirmations, and to the clinical experience of physicians. Not once was heard the voice of derision nor the words, "I do not believe it." If any one thought it, he did not say so.

The provings with dynamic medicines were studied to ascertain the harmony of the image and the consistency within its record of sensations, knowing that the proof of the proving is often within itself observed only by those who know, *never by agnostics.*

The President opened the meeting and read an able address full of useful thoughts and properly toned. The eulogy on the lamented Lippe was well chosen. The rulings were wise and the sayings from the Chair will ever be remembered.

The work of reporting papers lasted four days, and the enthusiasm was at the highest pitch.

The election of officers was most complimentary to the Association, as everybody wanted some one else to be President.

The election resulted as follows: Wm. A. Hawley, of Syracuse, N. Y., President; Wm. S. Gee, of Chicago, Vice-President; Samuel A. Kimball, of Boston, Secretary; J. D. Tyrrell, Toronto, Canada, Treasurer.

Next place of meeting, Toronto, Canada.

